# Publication Trends in Rehabilitative Effects of Acupuncture: A Visual Analysis of the Literature

**DOI:** 10.1155/2022/7705256

**Published:** 2022-04-11

**Authors:** Yanmei Zhong, Jihui Cao, Haizhen Lu, Zonghai Huang, Lu Liu, Chuanbiao Wen, Ji Chen

**Affiliations:** ^1^School of Medical Information Engineering, Chengdu University of Traditional Chinese Medicine, Chengdu 610075, China; ^2^Department of Orthopedics, Renshou County Hospital of Traditional Chinese Medicine, Chongqing 620500, China; ^3^Basic Medical School of Chengdu University of Traditional Chinese Medicine, Chengdu 610000, China; ^4^School of Acupuncture-Moxibustion and Tuina, Chengdu University of Traditional Chinese Medicine, Chengdu 610075, China; ^5^School of Foreign Languages, Chengdu University of Traditional Chinese Medicine, Chengdu 610075, China

## Abstract

**Objectives:**

To conduct a comprehensive analysis of scientific outputs in 2011–2021 regarding the rehabilitative effects of acupuncture on diseases.

**Methods:**

The study was conducted in the form of knowledge graph and data visualization, with data being drawn from the Web of Science Core Collection database.

**Results:**

Articles and reviews were the dominant types; China, Guangzhou University of Chinese Medicine and *Medicine* ranked was the active country, institution, and journal, respectively, in terms of issued articles. Systematic reviews and the meta-analyses of stroke and pain were extensively carried out in the past decade, whose principal interventions were manual acupuncture, electroacupuncture, scalp acupuncture, and dry needling correspondingly at Baihui (DU20) and Zusanli (ST36). And most frequently utilized rehabilitation assessment criteria were the Fugl-Meyer Assessment Scale and the Barthel Index. More recently, motor function and chronic obstructive pulmonary disease have captured researchers' attention, which might be the futuristic frontier.

**Conclusions:**

This article provided a relatively panoramic picture of the scientific outputs in acupuncture for disease rehabilitation, which may help readers embrace the heated topic and grasp the recent research focus on this field.

## 1. Introduction

Globally, the percentage of individuals undergoing functional decline is accelerating, primarily due to population aging, extended longevity, and the prevalence of noncommunicable diseases [1]. It is reported that around 15% of the world's population, more than 1 billion individuals, experience a certain form of disability, among them, 43,227,000 disability-stricken American people account for almost 13.2% of the US population [2, 3]. Estimates currently indicate that there are 2.4 billion people with health conditions that could benefit from rehabilitation; thus, the unmet need for rehabilitation is enormous [4, 5]. The International Classification of Functioning, Disability and Health (ICF) offers a valuable conceptual framework for rehabilitation, focusing on the 3 domains of physical function and structure, activity and participation, and the influence of environmental and personal factors. Essential elements of rehabilitation include an interdisciplinary approach, individualized rehabilitation programs, and the active participation of the patient. Acupuncture, one of the complementary alternative modalities, has been utilized in the health-care system to treat diseases all over the world and even achieves rehabilitative effects, such as pain, hypertension, arthritis, and stroke [6, 7]. Although extensive researches have been carried out on the benefits of acupuncture, no single study exists that demonstrates the states and concerns of acupuncture on disease convalescence. Therefore, our study aims to conduct a comprehensive analysis of scientific outputs in 2011–2021 about the rehabilitative effects of acupuncture on diseases.

Bibliometrics, a research method that applies mathematical and statistical methods to analyze written communication, can quantitatively and qualitatively evaluate scientific outputs in terms of countries, organizations, and topics. It has already been utilized as a method of providing insight into research in contemporary medicine and traditional Chinese medicine-related topics, such as yoga [8], acupuncture [9], Chinese medicinals [10], cancers [11], and cardiovascular diseases [12]. Web of Science Core Collection database (WoS), as one of the most comprehensive bibliographic sources available, frequently provides users an online access port to scientific literature with high quality, where we can get access to the cited references, one of the important indicators in bibliometrics. With the help of 2 bibliometric visualization tools, Citespace 5.8.R2 and VOSviewer 1.6.15, the knowledge graph of acupuncture for disease rehabilitation was depicted, and data visualization was carried out based on bibliometric analysis of annual publication trends, countries, authors, institutes, and research hotspots.

## 2. Materials and Methods

### 2.1. Search Strategy

The core collection of WoS has been searched to collate documents relating to acupuncture for rehabilitation. The search strategy covers acupuncture or its synonyms (Title or Topic) and rehabilitation or its synonyms (Title OR Topic). The publication period is limited to 2011 to 2021 (retrieved on October 25, 2021). Besides, there is no restriction to the article type, but the language is restricted to English (Table 1).

### 2.2. Inclusion Criteria

Referring to WHO's definition, rehabilitation is a set of interventions designed to optimize functioning and reduce disability in individuals with health conditions in interaction with their environment, and effective rehabilitation is as important as effective surgery to ensure functional independence [13]. Thus, any papers that mentioned the effects of acupuncture on optimizing functioning and reducing disability (such as mitigation of pain, dysphagia, restriction of movement) in individuals were included in our work. Two authors (Yanmei Zhong and Zonghai Huang) independently read the title and abstract of those 3766 pieces of papers and, any, disagreements were tackled by the third author (Jihui Cao). Consequently, 586 documents were included.

### 2.3. Bibliometric Analysis

#### 2.3.1. Key Bibliometric Analysis Terms

Knowledge graph visualization is a product of the development of graphical data technologies. Data visualization enables the integration of information into a single large network that contains semantic models of data for users to query and explore. In this way, raw data are transformed into graphical information, and the presentation of information is more figurative and intuitionistic.

Betweenness centrality refers to the number of times a node is located on the shortest route between other nodes, and it is one of the critical metrics to measure the importance of a node. The more times the node is located on the shortest paths, the higher the node centrality. It is generally considered that a node with a betweenness centrality greater than 0.1 is a critical node.

#### 2.3.2. Bibliometric Analysis Software

The bibliometric analysis and the visualization of the involved data have been conducted by 2 software: Citespace 5.8.R2 and VOSviewer 1.6.15. VOSviewer has been applied to present a network of authors, keywords, countries, institutes, as well as their density map, whose counting method was full counting. Citespace 5.8.R2 was utilized to depict the institute's cooperation network.

## 3. Results

### 3.1. Document Types and Publication Trend

**Figure 1:**
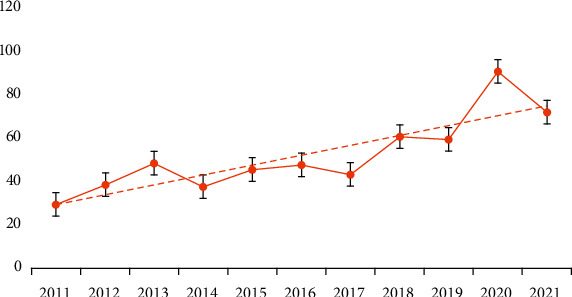
Annual publications trend.

The 586 papers involved are of 6 document types. The articles (415, 71%) were the most prevalent, followed by the reviews (152, 26%), the meeting abstracts (12, 2%), the editorial materials (4, 1%), the letters (2, 0%), and the collection (1, 0%). As shown in Figure 1, the overall publications in a decade exhibited a gradual upward trend; it increases following the function, *y* = 18.084ln(x) + 22.772, *R*^2^ = 0.5979, which is relatively stable. The publication number in 2019–2012 was the most noticeable one, followed by the number of articles issued in 2017-2018 and 2011–2013. The top 3 annual postings were 2020 (90), 2021 (71) and 2018 (60), indicating that the relevant literature has a trend for increase in recent years.

### 3.2. Analysis of Countries and Regions

**Figure 2:**
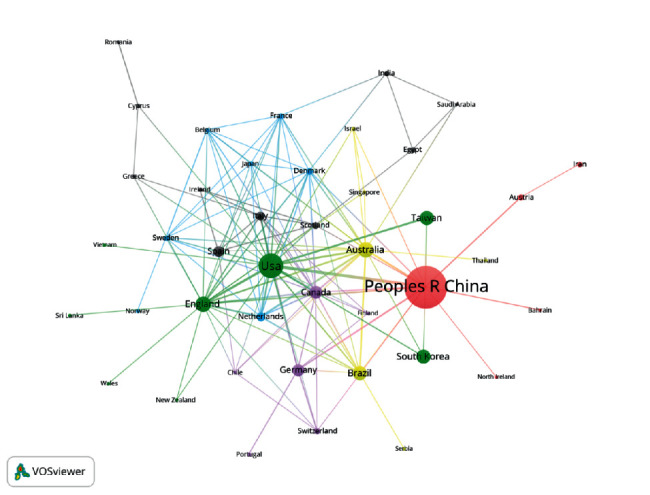
Co-authorship network of country.

The top 10 productive countries are presented in Table 2. As shown in Figure 2, there were 42 items, namely 42 countries constructed the cooperation network among each other. Among them, China occupied piles of documents relating to acupuncture on rehabilitation, with 15 links, followed by the USA which published 93 documents with 24 links with other countries. Besides USA (4002), Australia (2612), and England (2378) being the countries gaining major citations, they are also listed as the top 3 countries in terms of the number of articles issued in collaboration with other countries. Countries linked by colored line revealed that they have co-authorship. Admittedly, cross-country cooperation was obvious: The cooperation network was led by the USA, China, and Australia with mainly European countries, as only 6 countries (Malaysia, Qatar, Hungary, Mexico, Uzbekistan, and South Africa) had conducted the researchers independently.

### 3.3. Analysis of Institutions

Among the 970 institutions included, 12 institutions issued more than 10 articles. According to Table 3, Guangzhou University of Chinese Medicine ranked the top, and China Medical University and Beijing University of Chinese Medicine ranked the 2nd and 3rd, respectively. However, the studies carried out by the University of Sydney (1770), the University of Oxford (1996), and the University of Arizona (1592) were greatly recognized, whose publications accounted for only 7% of the total publications, with 12% overall citations. Accordingly, there is no imperative association between the quantity of the articles and their quality.

**Figure 3:**
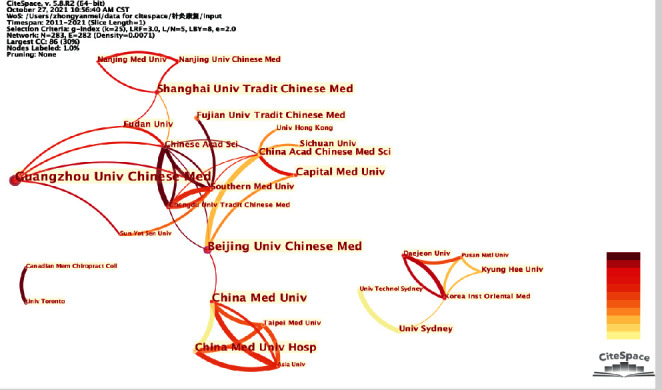
Institution cooperation network.

As shown in Figure 3, there were 3 major cooperation groups, which were led by the research team of Guangzhou University of Chinese Medicine, the China Medical University Hospital and the Korea Institute of Oriental Medicine. Admittedly, researches conducted by Chinese institutes were substantial in numbers, while the University of Sydney with only 7 documents received the most citations (1770), exerting significant academic impacts.

### 3.4. Analysis of Authors

**Figure 4:**
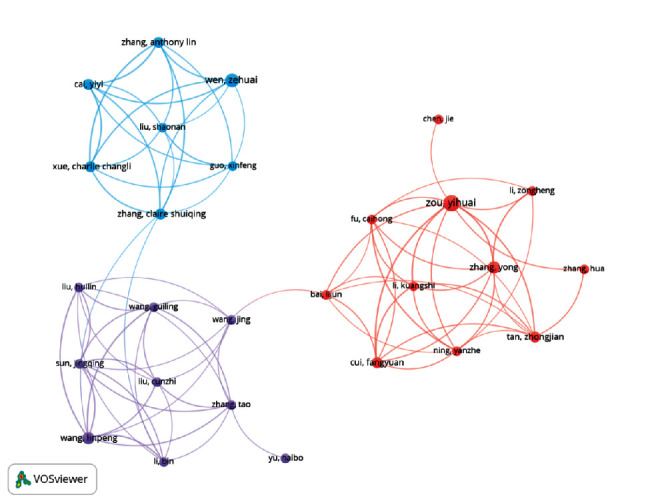
Author cooperation network map.

Lidian Chen, Jaunggeng Lin, and Yihuai Zou were the top 3 most productive authors (see Table 4), while Chou Roger with only 2 papers got 306 citations.  Table 5 presented the top 10 most co-cited authors, with Wu P., MacPherson, and Park J. correspondingly ranking the 1st, 2nd, and 3rd. MacPherson, Chou R., and Higgins J. P. T. all had the highest centrality, followed by Wu P. (0.12), Zhang S. H. (0.12), and Park J. (0.1). As revealed in Figure 4, there were 3 different colored clusters, indicating 3 major author teams.

### 3.5. Analysis of Journals

**Figure 5:**
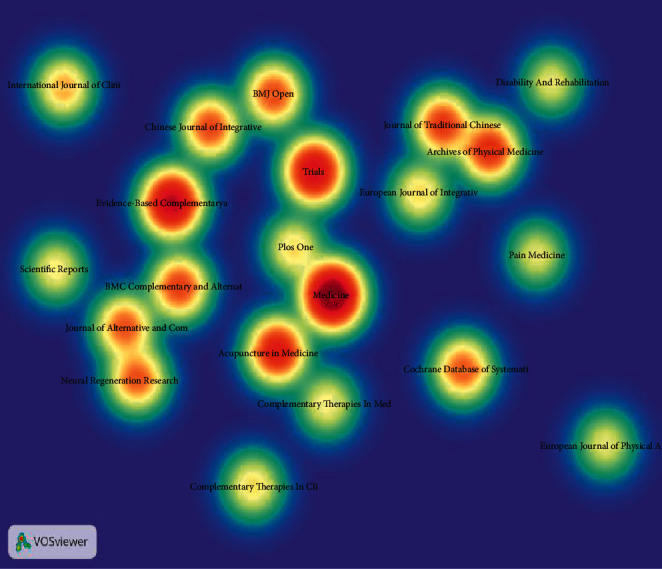
Density map of journals.

In density map, the warmer the node color, the more articles are released in the journal. As depicted in Figure 5, a multitude of articles has been released in *Medicine*, *Evidence-Based Complementary and Alternative Medicine*, and *Trials* (Table 6). *Cochrane Database of Systematic Reviews*, *Journal of Alternative and Complementary Medicine*, and *Archives of Physical Medicine and Rehabilitation* were the top 3 most referenced (Table 7). And *The Lancet* ranked the first in centrality (0.03).

Medicine focused on general internal medicine, whose publications covered serial topics such as stroke-induced balance dysfunction and central pain [14, 15], rehabilitation of methamphetamine addicts, neurogenic bladder, spinal cord injury, cerebral palsy, nausea, and vomiting after operation [16–19]. *Evidence-Based Complementary and Alternative Medicine* was inclined to focus on integrative and complementary medicine; it explored stroke-induced disorders, such as heart rate variability, motor dysfunction, paralysis, white matter impairment, shoulder pain [20–24], and chronic urinary retention due to spinal cord injury [25]. While papers from *Trials* consisted of protocols of research into experimental and clinical medicine, which covers chronic nonspecific low back pain, knee osteoarthritis, cavovarus foot, attention deficit, motor dysfunction, as well as aphasia and constipation induced by stroke [26–31].

### 3.6. Analysis of References

**Figure 6:**
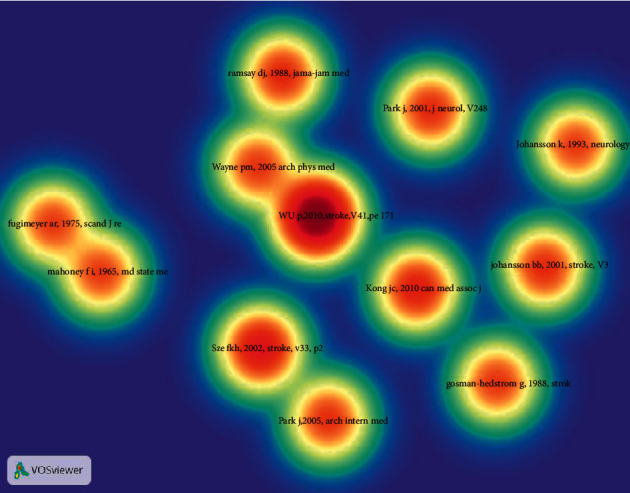
Density map of the co-cited references.

**Figure 7:**
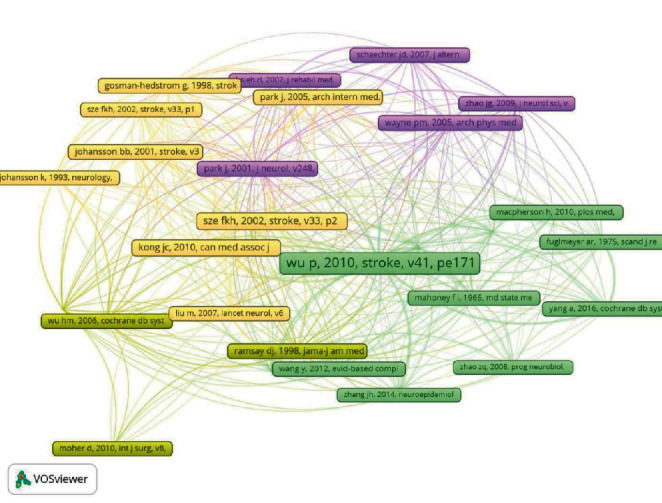
Network of the cited references.

The density visualization of the cited references has been illustrated in Figure 6, whose colors range from cool to warm. The color of Wu p, 2010, stroke, v41, pe171 was warmest, revealing that it was most credited, followed by Sze fkh, 2002, stroke, v33, p2604 and Kong jc, 2010, can med assoc j, v182, p1723. As shown in Table 8, Park. J's 2 articles occupied 2 places (Table 8). According to the network of co-cited references in Vosviewer, the relatedness of the references can be presented by the distance between 2 references or the color of the node. If 2 references are simultaneously cited by other references, to a certain degree, the 2 references share certain similarities. As depicted in Figure 7, the cited references can be divided into 4 major groups.

### 3.7. Analysis of Keywords

In total, 1164 keywords were identified by the VOSviewer. To achieve date cleaning, the thesaurus file of VOSviewer and the OpenRefine have been applied to merge synonyms (e.g., electro-acupuncture and electroacupuncture), merge words in the singular or plural form (e.g., clinical trial and clinical trials), and ignore relatively unimportant terms (e.g., humans and therapy). Besides, the minimum number of occurrences of the keywords was set as 5. As a result, 61 keywords have been analyzed.

#### 3.7.1. Analysis of Keywords by Co-Occurrence

**Figure 8:**
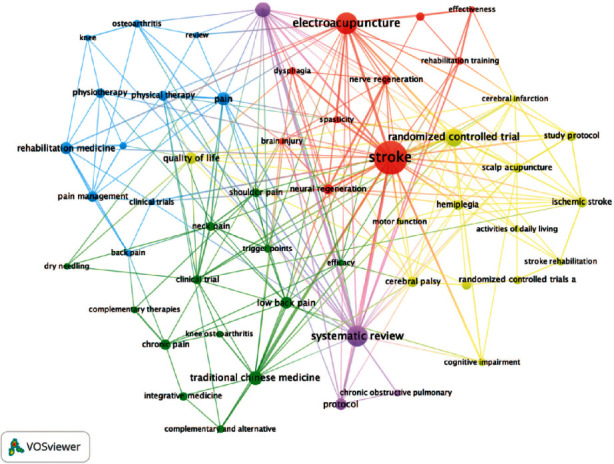
Network of keywords.

**Figure 9:**
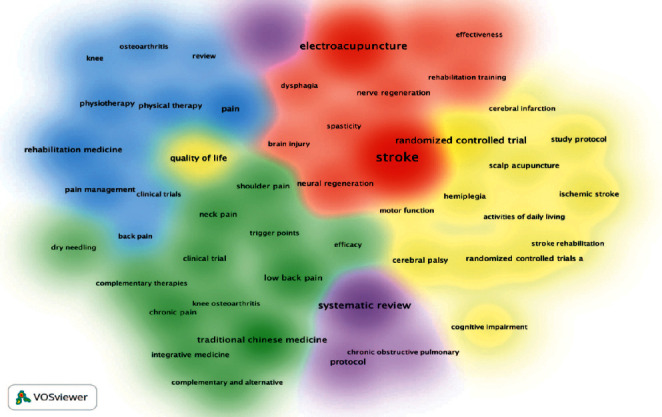
Density map of keywords.

As displayed in Figures 8 and 9, co-occurrent keywords can be grouped into 5 clusters, which are presented by “stroke” in the red cluster, “traditional Chinese medicine” in the green cluster, “systematic reviews” in the purple cluster, “randomized controlled trials” in the yellow cluster, and “pain” in the blue cluster. These keywords could be further categorized by disease, types of intervention, statistical analysis, and trial (Table 9).  Table 10 lists the top 10 keywords in terms of frequency and centrality, revealing that much work has been published regarding stroke, electroacupuncture, and systematic reviews. There is no denying that acupuncture research poured attention into stroke and pain rehabilitation whose statistical methods were systematic reviews and meta-analysis of randomized controlled trials, which is consistent with the abovementioned principal keyword categorization.

Consequently, acupoints for research of the stroke and the pain were summarized from corresponding literature with citations exceeding 5 (Supplementary files 1-2). Furthermore, the top 5 commonly utilized acupoints for the stroke and the pain were also summarized (Table 11). Admittedly, Baihui (DU20) was the indispensable acupoint for stroke convalescence, while the commonly utilized point for pain research was Zusanli (ST36). In addition, Quchi (LI11) and Zusanli (ST36) were both applied for the research of stroke and pain rehabilitation.

#### 3.7.2. Analysis of Keywords in Terms of Timeline

**Figure 10:**
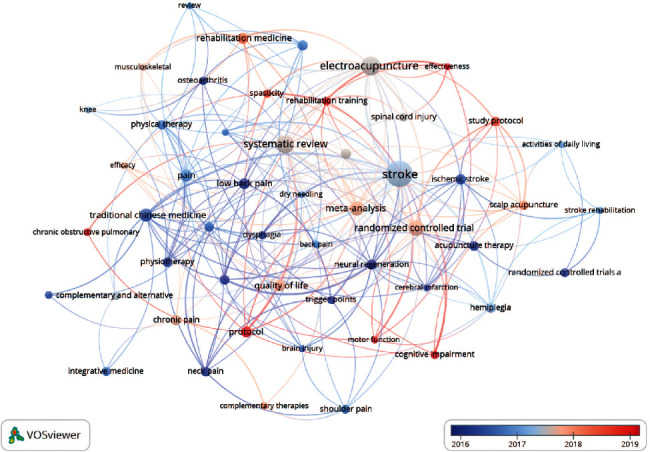
Overlay visualization of keywords.

Research concerns of acupuncture have evolved from low back pain, neural regeneration, and osteoarthritis in the primary stage to chronic obstructive pulmonary disease and cognitive impairment in recent years.  Figure 10 depicts the overlay visualization of keywords. When it comes to research of acupuncture for specific disease convalescence, based on the average publication year of keywords, initially, acupuncture was mainly employed for the rehabilitation of low back pain, osteoarthritis, dysphagia, etc. In the medium term, it was mainly indicated for cardiovascular and cerebrovascular diseases such as stroke and cerebral palsy, and more recently for cognitive impairment and chronic obstructive pulmonary disease, etc. (Table 12).

## 4. Discussion

### 4.1. Article Growth Curve

A considerable amount of highly credited literature was published in 2014; it was from 2014 that acupuncture rehabilitation-related articles issuance underwent a second surge in postings, which may be associated with OARSI guidelines for the nonsurgical management of knee osteoarthritis published by McAlindon et al. This guideline offered an “inconclusive” recommendation for acupuncture due to a lack of strong evidence for the acupuncture application and inconsistent conclusions of the American Rheumatology and AAOS guidelines for the treatment of arthritis [32]. This uncertain statement encouraged researchers to further explore the application of acupuncture and its clinical evidence, such as ischemic stroke, neck pain, dysphagia, and osteoarthritis. The second proliferation of papers may be related to the article published by Qaseem et al.; it recommended acupuncture as one of the nonpharmacological treatments for low back pain due to its moderate-quality evidence for chronic low back pain and low-quality evidence for most acute or subacute low back pain [33]. This finding greatly bolsters the research concerning acupuncture for pain. Considering that acupuncture has been gaining popularity in academic circles over the world, and the overall publications in a decade demonstrated a gradual upward trend, beyond the shadow of a doubt, corresponding publications are projected to increase.

### 4.2. General Performance on Acupuncture for Disease Rehabilitation

In the past decade, 49 countries made contributions to research on acupuncture for disease recovery. Although Chinese authors and institutes played an indispensable role in terms of cooperation and papers in quantity, it is the research conducted by US authors and institutions that are extensively appreciated. There are several reasons behind that: (1) With thousands of years of clinical practice, acupuncture has been more extensively accepted and recognized in China than in the others, likewise, the number of TCM scholars and institutions is in a superior position. (2) However, a slice of their data sources is too geographically specific to be representative, samples are not sufficient, and a multitude of their methodological design is not rationally designed. (3) Most high-quality journals are primarily in English, quite a few excellent papers may not be cited due to a host of them being in Chinese hindering their international spread. (4) The achievements made the US cannot separate from their rigorous work, whose experimental cycle was extended, and the geographical coverage was relatively widespread.

Although corresponding literature published by *Medicine*, *Evidence-Based Complementary and Alternative Medicine*, and *Trail* were overwhelming in quantity, there were also serial papers published in highly influential journals, such as *Cochrane Database of Systematic Reviews, Osteoarthritis and Cartilage, Annals of Internal Medicine*, etc. Those highly accredited articles are characterized by novel topics, sound design, rigorous implementation, scientific analysis, etc. Currently, an ocean of studies conducted by Chinese authors usually explore acupuncture's potential effects on convalescence. However, its mechanism is far from being sufficiently and conclusively investigated, and high-influential papers are inadequate with most papers' influence factors ranging from 1 to 3.

As for the top 10 most frequently cited references [34–43], 9 of them were meta-analyses of randomized trials, surprisingly, they all focused on acupuncture for stroke. However, their discussions on whether acupuncture was rewarding to stroke rehabilitation are quite controversial: 3 literature presented partial positive comments, 2 positive comments, and 4 negative comments. These conflicts might be attributed to their heterogeneous acupoints selection, inconsistent evaluation indicators, and varied treatment sessions. Although the controversy about acupuncture scientific evidence for strokes has raged unabated for years, there is a rough consensus among researchers that the assessment of acupuncture for stroke requires a well-designed trial with adequate samples and there is a pressing requirement to formulate a standard protocol for the treatment of stroke with acupuncture.

### 4.3. Acupuncture Types, Research Methods, and Statistical Analyses

#### 4.3.1. Acupuncture Types

Manual acupuncture is the technique of piercing the body's acupoints with needles. It is stated that acupuncture was a rewarding intervention, especially for neurological damages [44]. Acupuncture was effective in stroke, Parkinson's disease, Alzheimer's disease, and migraine [44–47]. Its mechanism can be contributed by the antioxidative, anti-inflammatory, and antiapoptotic effects. For instance, Lin Lu et al. found that acupuncture can modify neurological dysfunction and reduce brain edema in experimental ischemia through increased endogenous neurogenesis, including proliferation, migration, and differentiation of neural stem cells [48]. And the antiapoptotic role of acupuncture in the treatment of neurological diseases is manifested through alterations in the expression of Bcl-2, Bax, or caspases, which results in the regulation of mitochondrial or autophagic dysfunction, as well as the reduction of oxidative stress and inflammation [49].

Electroacupuncture (EA) utilizes both needle and electrical stimulation to combat or prevent diseases. It involves a micro-current close to the body's bioelectricity on the needles after the needles have been inserted into the acupoints and the sensation of qi arrives. It has been revealed that EA was a reliable treatment modality for acute ischemic stroke [50]. EA at Quchi (LI11) and Zusanli (ST36) had a neuroprotective effect against ischemic stroke, by inhibiting TLR4/NF-*κ*B-mediated inflammation [51]; EA at DU20 and DU24 improved cognitive impairment in rats with cerebral ischemia-reperfusion by upregulating Bcl-2 and downregulating Bax to counteract apoptosis [52]. Furthermore, EA could modulate central 5-HT and NA neuron function [53], whereas dense cranial electroacupuncture is productive in reducing depressive symptoms in stroke patients [54]. In addition, the combination of EA and regular care exhibits increasing potential to reduce poststroke spasticity of the overall and lower extremity [55] and EA might be successful in mitigating poststroke shoulder pain and better upper extremity function [56]. Considering motor dysfunction is frequently compliant with stroke, Jie Zhan et al. indicated that EA benefited the motor function of poststroke in variation in the Fugl-Meyer Assessment Scale [57].

Scalp acupuncture (SA) is also referred to as “neuroacupuncture.” It is performed by inserting filiform needles into the loose layer of scalp tissue to stimulate the brain neurons in the underlying area. Consequently, the primary indication of SA is encephalopathy; it is also a promising treatment of neurological disorders [58, 59]. Animal experiments have revealed that stimulation of specific areas of the scalp can increase the expression of PSD-95 and activate nitric oxide synthase [60], thereby improving learning memory capacity and intelligence, respectively [61, 62], and Chuen Heung Yau et al. suggested that SA could boost and initiate the release of neuronal transmitters, therefore contributing to the “reconnection” of defective neuronal pathways in autistic patients [63]. Hao Liu hypothesized that SA at Du20 and GB7 improved neurological function in rats with cerebral hemorrhage, possibly through the following mechanisms: restoration of microvascular integrity and enhancement of brain water content homeostasis [64]. Additionally, cerebral hemiplegic rats treated with scalp electroacupuncture exhibited improved behavioral scores, reduced apoptosis of hippocampal neurons, and higher expression levels of Akt and p-Akt, whose mechanism may be involved in the activation of the PI3K/Akt signaling pathway [65]. Besides, the therapeutic benefits of SA targeting vascular dementia have been substantiated by several empirical clinical studies [66–68]; SA also has the potential to be supplemented for patients presenting with serious adverse events or ineffective Parkinson's medications [69].

Dry needling (DN) refers to a procedure in which a fine filament needle is applied to the trigger point without the injection of any medications or water, whose primary applications are presented by myofascial pain syndrome and motor disorder, and it is accredited for its reduction of pain and muscle tension, improved range of motion, muscle strength, and coordination. As an example, DN had superiority in the alleviation of neck and shoulder pain in short and medium terms [70]. Lin Liu et al. approved that DN can significantly reduce lower back pain intensity [71]. However, the mechanism and effects of DN action fluctuate depending on the number of needles, the location and depth of the insertion, the intensity of the manipulation, and the stimulation of the local twitch response [72]. For example, a deeper needle insertion impacts the skin, fascia, and muscles and provides better analgesia than superficial stimulation [73–75]; when the needle is inserted 5 mm deep, the indirect effect on pain is oriented towards inhibiting the pain impulses in the C-fibers. Additionally, DN stimulates a local twitch response that reduces the presence of inflammatory mediators, pro-inflammatory cytokines, catecholamines, and neuropeptides within the MTRP, and this decrease is coupled with a diminished level of pain and palpable stiffness [73, 76–78]. It was also found that DN treatment outperformed myofascial release treatment in pain pressure thresholds; quality of life components related to physical role, bodily pain, vitality, and social functioning; and overall impact of fibromyalgia syndrome symptoms, sleep quality, anxiety status and trait, hospital anxiety, depression, overall pain intensity, and fatigue [79]. Furthermore, DN exerted a favorable effect on spasticity, dynamic stability, walking speed, self-reliance, and pain in patients with incomplete spinal cord injury [60].

#### 4.3.2. Research Method and Statistical Analyses

Randomized controlled trials were the preferred methods of study. However, not all reports adhered strictly to the CONSORT and STRICTA guidelines, and therefore the quality of reports of RCTs varied. Randomized control trials conducted in China, or recent examinations, usually yielded favorable results; investigations in recent years almost harbored the idea that acupuncture possessed a promising effect on disease rehabilitation [80–83]. As such, 10 RCTs involving 640 patients with brain tumors stated that only 2 interventions—personalized acupuncture with standard rehabilitation and family-based psychosocial intervention—produced improvements in health-related quality of life [84]. A review of 9 RCTs also noted the potentially favorable effects of acupuncture on dysphagia [85]. As for the systematic review and meta-analysis, after reading all the corresponding papers with citations over 10, they were dedicated to exploring the reliability and safety of acupuncture for pain and stroke.

### 4.4. Research Hotspots of Rehabilitative Effects of Acupuncture

Acupuncture for rehabilitation treatment is available for a multitude of disorders, which can be broadly divided into physical and mental disabilities. Among them, physical disabilities appeal to researchers, with stroke and pain occupying pivotal positions. There are several reasons behind this: their high prevalence, favorable clinical effects, and the cost-effectiveness of acupuncture.

#### 4.4.1. Stroke and Pain Disorder Mitigated by Acupuncture's Rehabilitative Effects

Stroke and pain disorders were the dominant diseases in acupuncture rehabilitation treatment. Epidemiological studies stated that disability after stroke is manifested as neurological deficits (e.g., motor, sensory, visual) and restricted ability to perform activities of daily living (ADL), and neuropsychological deficits (e.g., attention, memory, language) [86–88]. Accordingly, those disorders were also the concerns of acupuncture for stroke rehabilitation, and the outcomes of acupuncture for rehabilitation were represented by improvement or restoration function after stroke. For example, Ai Yang et al. maintained that for stroke patients in the subacute or chronic phase, acupuncture may mitigate neurological deficits, such as motor function, cognitive function, depression, swallowing function, pain, and spasticity. Moreover, there are no serious adverse events, when acupuncture is performed on stroke patients in the recovery period [89]. As for its mechanism, Lina Chavez et al. summarized it into 5 facets: (1) promoting cell proliferation in neurogenic areas and ischemic tissues, (2) regulating cerebral reflux in ischemic areas through the generation of vascular and vasoactive mediators, (3) regulating anti-apoptosis in ischemic areas, (4) regulating neurochemicals, and (5) increasing LIP in the hippocampal DG and CA1 areas [90]. For instance, it has been assumed that acupuncture can treat ischemic stroke through neurogenesis [91]; it influenced the cerebral blood flow in stroke patients, promoted the proliferation and differentiation of endogenous and ipsilateral hippocampal stem cells, reduced the expression of excitatory amino acids, and attenuated the cerebrovascular inflammatory response in poststroke treatment [92]. When it comes to poststroke cognitive impairment and depression, Caroline Yik-Fong Hung et al.'s meta-analysis revealed that acupuncture can be used to ameliorate cognitive function. In addition, acupuncture is found relatively more effective and safer than antidepressants in the treatment of poststroke depression [93].

Referring to a clinical guideline conducted by the American College of Physicians and the American Pain Society, acupuncture is one of the recommended treatments for lower back pain [94]; moreover, it can benefit individuals with subacute or chronic lower back pain in a cost-effective way [95]. Acupuncture can also achieve postoperative pain relief and decreased consumption of opioids [96]. The mechanism of acupuncture analgesia is mainly correlated with glial cells. (1) Electroacupuncture can inhibit glial cell activation by downregulating the chemokine CX3CL1 and increasing anti-inflammatory cells to achieve analgesic effects. Among them, an increase in anti-inflammatory cytokine, interleukin-10, can inhibit P38 mitogen-activated protein kinase and extracellular signal-regulated kinase pathway, which is associated with microglia activation of C-Jun N-terminal kinase signaling pathway and subsequent astroglial activation. (2) Acupuncture inhibits the release of various pain-related substances from glial cells: for example, the pro-inflammatory cytokines such as tumor necrosis factor alpha, interleukin-1 beta, interleukin-6, and prostaglandins. (3) Acupuncture stimulates the pain modulation system in the brain, including the anterior cingulate cortex, periaqueductal gray, and rostral extrapyramidal medulla [97].

*(1) Commonly Used Acupoints for Stroke and Pain*. Baihui (DU20) has been under the clinical practice of stroke treatment for over 2,000 years in China. According to TCM, it is located at the confluence of yang meridians, which is a vital point for the regulation of Qi movement and equilibrium of yin-yang. A meta-analysis revealed that acupuncture at DU20 manifested potential neuroprotective effects on stroke [98]. For example, EA at DU20 could mitigate brain edema and blood-brain barrier disruption caused by cerebral ischemia by reducing matrix metalloproteinase (MMP)-9 expression and activity; it also ameliorated motor performance and sensory function and was coupled with increased expression of vascular endothelial growth factor in ischemic brain tissue and peri-ischemic regions [99]. And laser acupuncture at DU20 could attenuate cognitive impairment and motor deficits in rats with ischemic stroke, whose specific mechanism may be attributed to increased neuronal survival in CA1 and CA3, increased GSH-Px and SOD activity, and decreased IL-6 to *β*-actin density ratio [100].

It was found that Zusanli (ST36) was indicated for visceral pain, arthritic pain, chronic knee pain, neck pain, low back pain, postoperative pain, neuropathic pain, inflammatory pain, and cancer pain. Its analgesic mechanism may be involved in the following mechanisms: (1) The mechanosensitive channel TRPV1 involved in acupuncture-related analgesia is highly expressive at ST36 [101]; acupuncture at ST36 attenuated cancer-induced pain by inhibiting the upregulation of TRPV1 mRNA and protein in DRGs [102]. (2) Collagen fibers in the ST36 could be the material basis for transmitting acupuncture analgesic information and participate in the transmission and conversion of acupuncture signals from acupuncture points to target organs [103]. (3) The production of analgesic effects of acupuncture may be correlated with the activation of certain chemicals by acupuncture stimulation. For instance, acupuncture at ST36 significantly increased the rate of local mast cell degranulation, which releases chemical substances such as substance p, histamine, and 5-hydroxytryptamine [104]. (4) EA at ST36 inhibited the expression of type I interleukin-1 receptor gene (IL-1RImRNA) in the cisternal area around the midbrain duct caused by peripheral inflammation, and the pain threshold was significantly increased.

EA at ST36 and Quchi (LI11) stimulated brain cell proliferation by activating the PI3K/Akt pathway and activating ERK signaling to reduce the infarct volume and the number of apoptotic nerve cells, respectively, thereby achieving neuroprotective effects against cerebral ischemic stroke [105]. It was also found that needling at the contralateral LI11 and ST36 probably facilitated motor-related activity in the lesioned hemisphere, increased blood volume and flow in the contralateral lobe, and modulated motor areas damaged by the stroke [106]. LI11 has been extensively utilized in China for shoulder pain and heat-induced disorders such as headache, sore throat, toothache, and ocular pain. However, far too little attention has been paid to the analgesic mechanism of LI11, while extensive research has been carried out on the analgesic effects of ST36.

#### 4.4.2. Novel Research on Acupuncture for Rehabilitation

“Motor function” and “chronic obstructive pulmonary disease” are emerging concerns in this field, as the abovementioned EA can mitigate motor function defects, one of the neurological impairments induced by stroke, by boosting VEGF levels in peripheral blood serum, cerebral blood flow, and cerebral blood volume [107]. Similar beneficial effects were also observed in DN, SA, and transcutaneous electrical point stimulation [108–110].

As for acupuncture's rehabilitation effects on chronic obstructive pulmonary disease (COPD), acupuncture may enhance the strength of the diaphragm in cases of COPD, thereby alleviating or preventing respiratory failure and improving the working capacity of the respiratory muscles. Acupuncture may also be useful in reducing hypoxia and carbon dioxide retention, whose mechanisms may be involved in the rehabilitation of diaphragmatic dysfunction in COPD by modulating the inflammatory response, oxidative stress, or neural respiratory drive [111]. According to a trial conducted by NGAI, in COPD patients, after 45 minutes of transcutaneous electrical nerve stimulation at the acupuncture points, there were significant increases in exertional expiratory volume and peak expiratory flow rate, as well as improvements in respiratory rate and dyspnea, all of which were associated with an increased level of *β*-endorphins [112]. In terms of COPD assessment test scores, the body acupuncture treatment together with the medication group outperformed the medication alone group, and hence acupuncture was positive to the health-related quality of life of COPD patients [113].

### 4.5. Commonly Utilized Rehabilitation Assessment Scales

It was concluded that commonly utilized rehabilitation assessment scales could be divided into 2 groups: scales for primary diseases mitigated by acupuncture's rehabilitative effects and general scales.

Scales for primary diseases: (1) Stroke rehabilitation: National Institutes of Health Stroke Scale (NIHSS) and Modified Rankin Scale. (2) Pain rehabilitation evaluation scales: Verbal Descriptor Scale (VDS), Faces Pain Scale-revised (FPS-R), Verbal Rating Scale (VRS), Visual Analogue Scale (VAS), and Numerical Rating Scale (NRS).

General scales: (It was concluded that the rehabilitative effects of acupuncture for diseases often involve the following indicators and corresponding scales): (1) Motor function: Fugl-Meyer Assessment Scale (FMA) and Brunnstrom. (2) The cognitive function: Mini-mental State Examination (MMES), Loewenstein Occupational Therapy Cognitive Assessment (LOTCA), and Montreal Cognitive Assessment (MoCA). (3) Activities of daily living: Barthel Index (BI), Modified Barthel Index (MBI), and Functional Independence Measure (FIM). (4) Dysphagy: Standardized Swallowing Assessment (SSA), Bedside Swallowing Assessment (BSA), and Videofluoroscopic Swallowing Study (VFSS). (5) Depression: Hamilton Rating Scale for Depression (HRSD) and Self-Rating Depression Scale (SDS).

### 4.6. Limitations

There are a few limitations to be considered in this study. On the one hand, although we included articles from the last decade of relevant literature, which helps readers to catch the recent hot topics and grasp latest update on acupuncture progress, for presenting a more comprehensive overview of acupuncture study, subsequent researchers may consider extending the literature inclusion time. On the other hand, although the WoS database is the most reliable publication and citation database and what we have indexed articles was from high impact journals, other relevant studies in PubMed, Scopus, and Google Scholar, etc. [114–116] could also be given consideration for incorporation into further studies.

## 5. Conclusions

This article provides a general overview of the research on the rehabilitative effects of acupuncture through bibliometric analysis of the publication trend, authors, organizations, cited references, and keywords in the form of knowledge graph and data visualization. The literature on acupuncture for rehabilitation showed an overall upward trend in a decade. Chinese authors and organizations published most papers, while American authors gain dominant citations. International cooperation was mainly fostered among developed countries.

Acupuncture was principally employed in stroke and pain rehabilitation, with interventions such as manual acupuncture, electroacupuncture, SA, and DN at Baihui and Zusanli. Currently, acupuncture for motor function and COPD has captured researchers' attention, which might be the futuristic frontier. Relevant studies were based on meta-analyses or systematic reviews. The conflicts of meta-analyses of randomized controlled trials could be attributed to small samples, heterogeneous acupoints selection, inconsistent evaluation indicators, and varied treatment sessions and methodological flaws, such as incomplete assessment of bias, inconsistent assessment of randomization, allocation concealment, and blinding [117]. Besides, current evidence points to far more complex mechanisms; to better explore the mechanisms of analgesia and neurological effect of acupuncture, standard guidelines should be developed and applied in research, which elaborates on the criteria for point selection, clarifying the rehabilitation rating scale, and specifying the treatment course.

## Figures and Tables

**Table 1:** Search strategy.

Id	Result	Search strategy
^#^1	110,370	TS = (acupuncture OR needling OR acupuncture-moxibustion OR electroacupuncture OR moxibustion OR hydro-acupuncture OR acupoint OR warming needle OR ear-acupuncture OR scalp-acupuncture OR pharmacopuncture OR acupuncture therapy OR thread embedding therapy OR auricular acupuncture) OR TI = (Acupuncture OR needling OR acupuncture-moxibustion OR electroacupuncture OR moxibustion OR hydro-acupuncture OR acupoint OR warming needle OR ear-acupuncture OR scalp-acupuncture OR pharmacopuncture OR acupuncture therapy OR thread embedding therapy OR auricular acupuncture)

^#^2	710,534	TS = (rehabilitation OR convalescence OR healing OR recovery, OR recuperation) OR TI = (rehabilitation OR convalescence OR healing OR recovery OR recuperation)

^#^3	3766	^#^1 and ^#^2

**Table 2:** The top 10 most productive countries or regions.

Rank	Country or regions	Document	Centrality
1	China mainland	279	0.35
2	USA	93	0.46
3	Australia	38	0.21
4	England	37	0.21
5	South Korea	32	0.00
6	Taiwan	31	0.00
7	Brazil	28	0.07
8	Canada	24	0.13
9	Germany	24	0.09
10	Spain	18	0.02

**Table 3:** The top 10 most productive institutions.

Rank	Document	Institution	Centrality	Citations
1	28	Guangzhou Univ Chinese med	0.02	116
2	21	China med Univ	0.08	267
3	21	Beijing Univ Chinese med	0.10	133
4	20	Shanghai Univ tradit Chinese med	0.03	84
5	18	China med Univ hosp	0.03	252
6	14	Capital med Univ	0.03	122
7	12	Fujian Univ tradit Chinese med	0.01	251
8	11	China acad Chinese med sci	0.06	121
9	9	Sichuan Univ	0.01	253
10	9	Chinese acad sci	0.05	33

**Table 4:** The top 10 most productive authors.

Rank	Documents	Author	Citations
1	9	Lidian Chen	162
2	9	Jaunggeng Lin	140
3	9	Yihuai Zou	73
4	7	Jing Tao	140
5	6	Liwei Chou	96
6	6	Shanli Yang	61
7	6	Zehuai Wen	46
8	5	Taliang Chen	63
9	5	Hsinlong Lane	63
10	5	ChienChang Liao	63

**Table 5:** The top 10 co-cited authors.

Rank	Cited author	Citations	Centrality
1	Wu P.	71	0.12
2	MacPherson	70	0.13
3	Park J.	57	0.10
4	Moher D.	56	0.09
5	Sze F. K. H.	52	0.04
6	Zhang S. H.	52	0.12
7	Chou R.	47	0.13
8	Higgins J. P. T.	45	0.13
9	Furlan A. D.	44	0.08
10	Wang Y.	42	0.06

**Table 6:** The top 10 most productive journals.

Rank	Journal	Documents	Citations	Average citations	Influence factor
1	Medicine	44	97	2.2	1.889
2	Evid-based compl alt	31	144	4.6	2.630
3	Trials	28	139	5.0	2.279
4	Acupunct med	22	209	9.5	2.266
5	Arch phys med rehabil	18	519	28.8	3.996
6	J tradit chin med	16	121	7.6	0.848
7	BMC complem altern med	15	257	17.1	3.476
8	BMJ open	14	67	4.8	2.692
9	Chin j integr med	14	158	11.3	1.978
10	Cochrane db syst rev	13	634	48.8	9.289

**Table 7:** The top 10 cited journals.

Rank	Cited journal	Citations	Centrality	Influence factor
1	Cochrane db syst rev	213	0.02	9.289
2	J altern complem med	212	0.02	2.582
3	Arch phys med rehab	210	0.01	3.966
4	Stroke	205	0.02	7.914
5	Evid-based compl alt	193	0.01	2.630
6	Acupunct med	163	0.01	2.267
7	Lancet	160	0.03	79.323
8	PLoS One	156	0.01	3.240
9	Pain	151	0.02	6.961
10	Zhongguo zhen jiu	148	0.01	1.650

**Table 8:** The top 10 most cited references.

Rank	Cited references	Citations	Doi
1	Wu p, 2010, stroke, v41, pe171	63	10.1161/strokeaha.109.573576
2	Sze fkh, 2002, stroke, v33, p2604	34	10.1161/01.str.0000035908. 74261.c9
3	Kong jc, 2010, can med assoc j, v182, p1723	28	10.1503/cmaj.091113
4	Johansson bb, 2001, stroke, v32, p707	24	10.1161/01.str.32.3.707
5	Park j, 2001, j neurol, v248, p558	24	10.1007/s004150170132
6	Park j, 2005, arch intern med, v165, p2026	23	10.1001/archinte.165.17.2026
7	Gosman-hedstrom g, 1998, stroke, v29, p2100	22	10.1161/01.str.29.10.2100
8	Wayne pm, 2005, arch phys med rehab, v86, p2248	22	10.1016/j.apmr.2005.07.287
9	Johansson k, 1993, neurology, v43, p2189	21	10.1212/wnl.43.11.2189
10	Liu m, 2007, lancet neurol, v6, p456	20	10.1016/s1474-4422(07)70004-2

**Table 9:** Summary table of keywords.

Classification	Keywords		Frequency
Diseases	Stroke	Dysphagia	19
		Quality of life	14
		Neural regeneration	11
		Hemiplegia	10
		Cognitive impairment	7
		Spasticity	7
		Activities of daily living	5
		Motor function	5
	Pain	Low back pain	48
		Chronic pain	9
		Neck pain	8
		Shoulder pain	8
	Spinal cord injury		13
	Cerebral palsy		11
	Osteoarthritis		6
	Chronic obstructive pulmonary disease		5
	Brain injury		5

Types of intervention	Manual acupuncture		151
	Electroacupuncture		40
	Scalp acupuncture		10
	Dry needling		5

Statistical analyses	Systematic review		68
	Meta-analysis		61

Trials	Randomized controlled trials		36
	Clinical trial		9

**Table 10:** The top 10 keywords in terms of frequency and centrality.

Rank	Keywords	Frequency	Centrality
1	Stroke	115	0.13
2	Electroacupuncture	79	0.11
3	Systematic review	68	0.11
4	Randomized controlled trial	65	0.11
5	Meta-analysis	61	0.10
6	Pain	54	0.10
7	Low back pain	47	0.80
8	Protocol	47	0.70
9	Cerebral palsy	42	0.06
10	Chronic pain	36	0.09

**Table 11:** The top 5 acupoints for stroke and pain.


Acupoints	Frequency
*Stroke*	
Baihui (DU20)	46
Sanyinjiao (SP6)	20
Quchi (LI11)	19
Zusanli (ST36)	18
Hegu (LI14)	17

*Pain*	
Acupoints	Frequency
Zusanli (ST36)	48
Zhongfeng (LI4)	21
Yanglingquan (GB34)	18
Huantiao (GB30)	10
Quchi (LI11)	9

**Table 12:** Acupuncture for specific disease rehabilitation.

Keywords	Frequency	Average publication year
Low back pain	13	2,014
Osteoarthritis	7	2,015
Dysphagia	7	2,015
Neck pain	9	2,016
Ischemic stroke	11	2,016
Cerebral infarction	5	2,016
Knee osteoarthritis	6	2,016
Brain injury	5	2,016
Shoulder pain	8	2,017
Back pain	6	2,017
Pain	16	2,017
Stroke rehabilitation	6	2,017
Hemiplegia	10	2,017
Stroke	83	2,017
Spinal cord injury	8	2,018
Cerebral palsy	11	2,018
Chronic pain	11	2,018
Spasticity	7	2,019
Cognitive impairment	7	2,019
Chronic obstructive pulmonary disease	6	2,020

## Data Availability

Data covered in this article can be gathered from the Web of Science.

## Supplementary Materials

Supplementary Materials Supplementary file 1 is the summary of acupoints for stroke, and supplementary file 2 is the summary of acupoints for pain.
